# DEB-TACE-HAIC combined with donafenib and camrelizumab in the treatment of unresectable hepatocellular carcinoma: a multicenter retrospective study

**DOI:** 10.3389/fimmu.2026.1779170

**Published:** 2026-05-29

**Authors:** Yuanjin Ma, Jian Long, Shangbo Shi, Xinyi Fang, Ruidong Wang, Feng Xiong, Hong Ren, Wei Li, Xuexian Zhang

**Affiliations:** 1 Department of Interventional Medicine, The Second Affiliated Hospital of Wenzhou Medical University, Wenzhou, Zhejiang, China; 2 Department of Vascular and Interventional Radiology, Jingmen People’s Hospital, Jingchu University of Technology Affiliated Jingmen People’s Hospital, Jingmen, Hubei, China; 3 Department of Interventional Medicine, The First Affiliated Hospital of Guangzhou Medical University, Guangzhou, Guangdong, China; 4 Department of Interventional Radiology, The First Affiliated Hospital of Kunming Medical University, Kunming, Yunnan, China; 5 Department of Interventional Radiology, Qujing Second People’s Hospital, Qujing, Yunnan, China

**Keywords:** camrelizumab, donafenib, drug-eluting bead transarterial chemoembolization, hepatic arterial infusion chemotherapy, hepatocellular carcinoma

## Abstract

**Objective:**

To evaluate the efficacy and safety of drug-eluting bead transarterial chemoembolization (DEB-TACE) combined with hepatic arterial infusion chemotherapy (HAIC), donafenib and camrelizumab in the treatment of unresectable hepatocellular carcinoma (uHCC).

**Methods:**

This multicenter retrospective analysis enrolled patients with unresectable hepatocellular carcinoma (uHCC) from 4 Grade A tertiary hospitals in China, with data collected between December 2022 and December 2024. Patients were stratified into two groups based on their treatment modalities: the DEB-TACE-HAIC combined with donafenib and camrelizumab group (DHDC) and the DEB-TACE combined with donafenib and camrelizumab group (DDC). The modified Response Evaluation Criteria in Solid Tumors (mRECIST) was utilized to assess and compare the objective response rate (ORR) and disease control rate (DCR) between the two cohorts. Survival curves were generated using the Kaplan-Meier method, and differences in progression-free survival (PFS) and overall survival (OS) were analyzed by the Log-rank test. Additionally, the Cox proportional hazards regression model was employed to identify independent prognostic factors for PFS and OS.

**Results:**

A total of 122 patients were ultimately enrolled in this study, with 58 patients were enrolled in the DHDC group and 64 patients were enrolled in the DDC group. Baseline characteristics were well balanced between the two groups. The median follow-up duration was 18 months in the DHDC group and 14 months in the DDC group. Before inverse probability of treatment weighting (IPTW) adjustment, the DHDC group had higher ORR (74.1% vs. 51.6%, P = 0.010) and DCR (91.4% vs. 75.0%, P = 0.017) than the DDC group. These advantages remained after IPTW adjustment (ORR: 72.5% vs. 53.4%, P = 0.036; DCR: 90.7% vs. 75.3%, P = 0.033). Kaplan–Meier analysis showed that the DHDC group had longer median PFS (16 vs. 11 months, P < 0.001) and OS (28 vs. 18 months, P < 0.001) than the DDC group before IPTW adjustment; these differences remained after IPTW (PFS: 16 vs. 11 months, P < 0.001; OS: 28 vs. 19 months, P < 0.001).On multivariable Cox analysis, portal vein tumor thrombosis (PVTT) (*P* = 0.007) and extrahepatic metastasis (*P* < 0.001) were identified as independent risk factors for PFS, whereas treatment with the DHDC regimen was independently associated with improved PFS (*P* < 0.001). Similarly, PVTT (*P* = 0.016) and extrahepatic metastasis (*P* < 0.001) were independent predictors of poorer OS, while DHDC treatment remained an independent protective factor for OS (*P* < 0.001). Subgroup analyses consistently demonstrated clinically meaningful improvements in both PFS and OS with DHDC therapy across most prespecified clinical subgroups. Notably, more pronounced survival benefits were observed among male patients, those with hepatitis B virus infection, multiple tumors (>3), underlying cirrhosis, PVTT, extrahepatic metastasis, Barcelona Clinic Liver Cancer stage (BCLC) C disease, and tumor diameter greater than 10 cm. Although the DHDC group experienced a higher incidence of nausea and vomiting compared with the DDC group (*P* < 0.05), these adverse events were limited to grade 1–2 severity. No significant differences were observed between the two groups with respect to the overall incidence or severity of other adverse events (*P* > 0.05).

**Conclusion:**

Among patients with uHCC, the DHDC quadruple regimen was associated with improved survival outcomes while maintaining an acceptable safety profile. These findings suggest that a treatment strategy incorporating DEB-TACE–HAIC in combination with targeted therapy and immunotherapy may represent a more effective therapeutic option for selected patients with uHCC, warranting further prospective investigation.

## Introduction

Primary liver cancer ranks as the sixth most commonly diagnosed malignancy worldwide and represents the third leading cause of cancer-related mortality (1). Hepatocellular carcinoma (HCC) is the predominant histologic subtype of primary liver cancer, accounting for approximately 75% to 85% of cases (2). At the time of diagnosis, nearly 70% of patients with HCC present with advanced-stage disease and are no longer candidates for curative surgical resection (3).

Transarterial chemoembolization (TACE) has therefore been established as a cornerstone therapeutic option for patients with unresectable hepatocellular carcinoma (uHCC). Currently, TACE can be broadly classified into conventional TACE (cTACE) and drug-eluting bead TACE (DEB-TACE), each differing in drug delivery and embolization characteristics (4). Conventional transarterial chemoembolization (cTACE) involves the intra-arterial administration of an emulsion of iodized oil and chemotherapeutic agents, followed by embolic materials, to target hepatocellular carcinoma. Although cTACE has demonstrated clinical efficacy, its therapeutic performance is limited by the high fluidity of iodized oil and the lack of controlled drug release. These limitations may result in inconsistent embolization, a relatively short duration of intratumoral drug retention, and an increased risk of systemic adverse effects (5). In contrast, DEB-TACE employs microspheres that are loaded with chemotherapeutic agents through an ion-exchange mechanism and subsequently delivered directly to the tumor-feeding arteries via catheterization. The drug-eluting beads not only achieve permanent embolization of tumor-supplying vessels but also enable sustained, localized release of chemotherapy within the tumor tissue. This pharmacokinetic profile allows higher intratumoral drug concentrations while reducing systemic exposure, thereby potentially improving treatment safety (6). Accumulating evidence suggests that, compared with cTACE, DEB-TACE is associated with more favorable tumor responses, prolonged survival outcomes, and an improved safety profile (7–9).

Hepatic arterial infusion chemotherapy (HAIC) has emerged as a potentially effective therapeutic approach for patients with advanced hepatocellular carcinoma. As a locoregional treatment modality, HAIC enables the direct delivery of anticancer agents to tumor tissues through catheter-based infusion via the hepatic artery (10).Although TACE and HAIC represent distinct forms of locoregional therapy, accumulating evidence indicates that their combined application may further enhance therapeutic outcomes in patients with unresectable hepatocellular carcinoma (11–13).

Sorafenib was the first molecularly targeted agent approved for the treatment of unresectable or metastatic hepatocellular carcinoma and was previously considered a standard first-line systemic therapy (14). Donafenib is a novel orally administered small-molecule multikinase inhibitor that targets vascular endothelial growth factor receptors (VEGFRs), platelet-derived growth factor receptors (PDGFRs), and multiple Raf kinases, thereby inhibiting tumor cell proliferation and angiogenesis.

As a deuterated derivative of sorafenib, donafenib contains a deuterated N-methyl moiety, a structural modification that enhances molecular stability and improves its pharmacokinetic profile (15). In a phase II–III clinical trial, donafenib was associated with superior overall survival outcomes compared with sorafenib in patients with unresectable hepatocellular carcinoma (16).

In recent years, immune checkpoint inhibition targeting programmed cell death protein 1 (PD-1) has achieved substantial advances, demonstrating promising clinical activity in the treatment of hepatocellular carcinoma (17). Camrelizumab, a PD-1 inhibitor, has been approved by the National Medical Products Administration of China for the treatment of HCC (18).

Growing evidence suggests that the combination of immunotherapy with antiangiogenic agents may exert synergistic antitumor effects, leading to improvements in progression-free survival and overall survival among patients with HCC (19–21). Accordingly, integrated treatment strategies combining locoregional and systemic therapies have become a central component of clinical management for patients with unresectable hepatocellular carcinoma in China (22).

To date, evidence regarding the combination of targeted therapy and immunotherapy on the basis of DEB-TACE–HAIC for the treatment of uHCC remains limited. Accordingly, the present study was conducted to evaluate the efficacy and safety of a treatment strategy combining DEB-TACE–HAIC with donafenib and camrelizumab in patients with uHCC.

## Materials and methods

### Study design and patient selection

This study was designed as a multicenter, retrospective analysis of patients with unresectable hepatocellular carcinoma treated between December 2022 and December 2024 at four tertiary hospitals in China. Baseline demographic and clinical characteristics were collected, including sex, age, hepatitis B virus infection status, presence of cirrhosis, Child–Pugh class, Eastern Cooperative Oncology Group performance status (ECOG PS), alpha-fetoprotein (AFP) level, maximum tumor diameter, tumor number, Barcelona Clinic Liver Cancer (BCLC) stage, presence of portal vein tumor thrombosis, and extrahepatic metastasis.

Patients were categorized into two groups according to whether hepatic arterial infusion chemotherapy was administered: the DHDC group, which received DEB-TACE–HAIC in combination with donafenib and camrelizumab, and the DDC group, which received DEB-TACE combined with donafenib and camrelizumab without HAIC. As this was a retrospective study, treatment allocation was not randomized. The choice between DHDC and DDC regimens was determined through a shared decision-making process between physicians and patients, based on individual patient characteristics (e.g., tumor burden, vascular invasion, extrahepatic metastasis, liver function, and performance status), as well as patient preferences, treatment tolerance, and economic considerations.

The study protocol was approved by the institutional review boards of the participating centers. Given the retrospective, noninterventional nature of the study and the use of fully deidentified data, the requirement for informed consent was waived. All procedures were conducted in accordance with the principles of the Declaration of Helsinki.

Patients were enrolled according to predefined inclusion and exclusion criteria. The inclusion criteria were as follows (1): age between 18 and 80 years (2); a clinical or histopathologic diagnosis of unresectable hepatocellular carcinoma (3); an Eastern Cooperative Oncology Group performance status score of 0 or 1 (4); preserved liver function with a Child–Pugh class of A or B (5); the presence of at least one measurable intrahepatic lesion (6); and receipt of either DEB-TACE–HAIC combined with donafenib and camrelizumab (DHDC) or DEB-TACE combined with donafenib and camrelizumab (DDC). The exclusion criteria were as follows (1): the presence or a history of autoimmune disease, or prior use of immunosuppressive agents;(2) any previous exposure to targeted therapy or immunotherapy;(3) a history of locoregional treatments, including hepatic resection, tumor ablation, transarterial chemoembolization (TACE), or hepatic arterial infusion chemotherapy (HAIC);(4) hepatocellular carcinoma concomitant with other malignancies; (5) incomplete clinical data.

### Treatment protocol

#### DEB-TACE procedure

All DEB-TACE procedures were performed by experienced senior interventional radiologists. Briefly, a 5-French Yashiro catheter (Terumo, Japan) was introduced via the femoral artery and advanced to the celiac trunk for angiography, followed by selective hepatic arteriography to identify tumor-feeding arteries. When vascular visualization was inadequate or tumor staining was incomplete in certain hepatic regions, selective angiography of the superior mesenteric artery, left gastric artery, and inferior phrenic artery was performed to detect ectopic hepatic arterial branches or extrahepatic collateral vessels supplying the tumor. Subsequently, a 2.7-F RAPIDTHRU microcatheter (Jiangsu Hengrui Pharmaceuticals, China) was selectively advanced into each tumor-feeding artery for embolization. Under fluoroscopic guidance, CalliSpheres drug-eluting beads (diameter, 100–300 μm; Jiangsu Hengrui Pharmaceuticals, China) loaded with 50 mg of epirubicin hydrochloride were slowly infused into the tumor-feeding arteries at a rate of approximately 1 mL per minute. If residual tumor staining persisted after the administration of one vial of CalliSpheres beads, additional embolization was performed using unloaded 8Spheres microspheres (diameter, 300–500 μm; Jiangsu Hengrui Pharmaceuticals, China) until the embolization endpoint was achieved. The embolization endpoint was defined as near stasis of blood flow in the tumor-feeding arteries. In patients with a high tumor burden, including those with large or multifocal tumors, a staged embolization strategy was adopted to reduce the risk of hepatic failure. In such cases, embolization was completed over two to three sessions, with the final session achieving the predefined embolization endpoint.

#### HAIC procedure

In patients assigned to the DHDC group, following completion of the DEB-TACE procedure, the microcatheter was left *in situ* within the proper hepatic artery, left hepatic artery, or right hepatic artery. After returning to the ward, continuous hepatic arterial infusion chemotherapy was administered through the indwelling microcatheter. HAIC was delivered using a modified FOLFOX regimen (23). Specifically, oxaliplatin was administered at a dose of 85 mg per square meter via arterial pump infusion over 2 to 3 hours, followed by leucovorin at a dose of 400 mg per square meter administered intravenously over 1 hour, and fluorouracil at a dose of 400 mg per square meter administered as an intra-arterial bolus. This was followed by continuous intra-arterial infusion of fluorouracil at a dose of 2400 mg per square meter over 46 hours. When follow-up contrast-enhanced computed tomography or magnetic resonance imaging demonstrated residual viable tumor or intrahepatic recurrence, DEB-TACE–HAIC or DEB-TACE was repeated at intervals of 3 to 4 weeks, depending on hepatic functional reserve.

#### Donafenib therapy

Donafenib was initiated within one week after the DEB-TACE procedure and administered orally at a dose of 200 mg twice daily. When repeat DEB-TACE was scheduled, donafenib was withheld for three days before the procedure. During donafenib treatment, drug-related adverse events were closely monitored. Patients who experienced grade 1 or 2 adverse events continued treatment at the initial dose with intensified clinical monitoring. For patients who developed grade 3 or higher adverse events, the dose of donafenib was reduced to 200 mg once daily. If adverse events remained intolerable after dose reduction, donafenib was permanently discontinued.

#### Camrelizumab therapy

Camrelizumab (Jiangsu Hengrui Pharmaceuticals, China) was initiated within one week after the DEB-TACE procedure. The drug was administered intravenously at a dose of 200 mg over 30 minutes every three weeks, with repeated dosing according to the treatment cycle. If immune-related serious adverse events occurred during treatment, camrelizumab was immediately discontinued, and appropriate immunosuppressive therapy was administered based on the severity of the adverse event and the organ systems involved.

### Follow−up and response assessment

All patients underwent routine follow-up assessments every 3 to 4 weeks, including contrast-enhanced computed tomography or magnetic resonance imaging of the liver, electrocardiography, laboratory evaluations (including biochemical tests), complete blood counts, coagulation profiles, liver function tests, serum alpha-fetoprotein levels, and assessments of clinical symptoms and physical signs. The primary end points were progression-free survival (PFS) and overall survival (OS). PFS was defined as the interval from the initiation of treatment to disease progression, and OS was defined as the interval from treatment initiation to death from any cause or the date of the last follow-up assessment. Secondary end points included objective response rate (ORR), disease control rate (DCR), and safety. Tumor response and disease progression were evaluated according to the modified Response Evaluation Criteria in Solid Tumors (mRECIST) (24). Post-treatment tumor responses were categorized as complete response (CR), partial response (PR), stable disease (SD), or progressive disease (PD). ORR was defined as the proportion of patients who achieved CR or PR, and DCR was defined as the proportion of patients who achieved CR, PR, or SD.

Treatment-related adverse events were assessed and graded according to the Common Terminology Criteria for Adverse Events, version 5.0 (25). The data cutoff date for follow-up was December 2025.

### Statistical analysis

Statistical analyses were performed using SPSS software, version 25.0 (IBM, New York, USA), and R software, version 4.2.2. Categorical variables were summarized as counts and percentages and compared using the chi-square test or Fisher’s exact test, as appropriate. Survival curves were generated using the Kaplan–Meier method, and differences between groups were assessed with the log-rank test. Overall survival was estimated using the Kaplan–Meier method, and patients who were alive at the data cutoff date were treated as censored observations. Univariable and multivariable Cox proportional-hazards regression models were applied to identify factors associated with PFS and OS, with results expressed as hazard ratios (HRs) and corresponding confidence intervals (CIs). Variables with a *P* value of less than 0.10 in univariable analyses were entered into the multivariable models. A two-sided *P* value of less than 0.05 was considered to indicate statistical significance. To enhance the robustness of the study results and address potential confounding bias, we employed inverse probability of treatment weighting (IPTW) to emulate randomization.

## Results

### Patient characteristics

**Figure 1:**
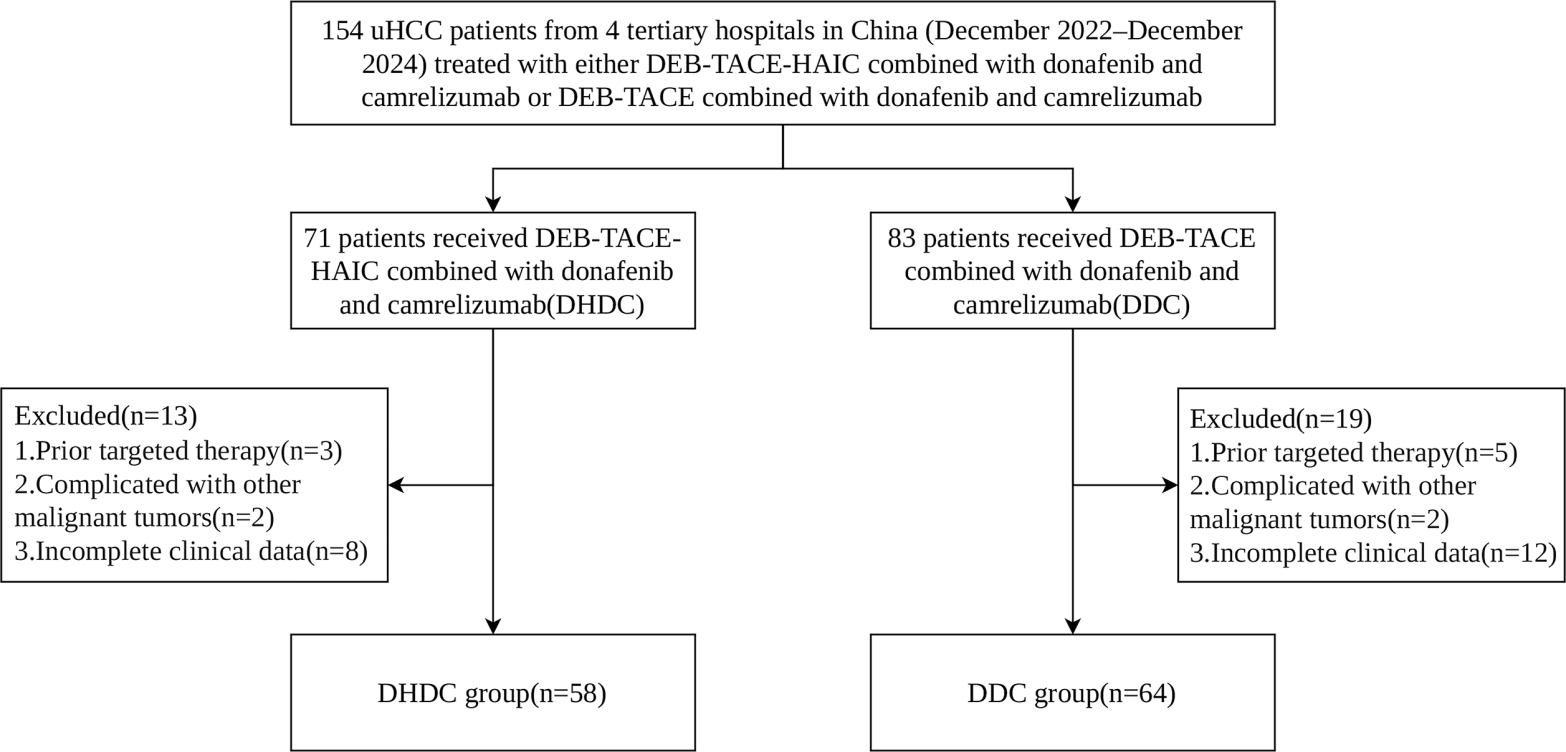
Patient screening flow chart. DEB-TACE, drug-eluting bead transarterial chemoembolization; HAIC, hepatic arterial infusion chemotherapy; DHDC, DEB-TACE-HAIC combined with donafenib and camrelizumab; DDC, DEB-TACE combined with donafenib and camrelizumab.

During the study period, 154 patients with uHCC who received DEB-TACE–HAIC combined with donafenib and camrelizumab or DEB-TACE combined with donafenib and camrelizumab were assessed for eligibility. Of these, 32 patients were excluded because they did not meet the inclusion criteria. Ultimately, 122 patients were included in the analysis. The patient selection process is illustrated in **Figure 1**. Among the included patients, 58 were assigned to the DHDC group and 64 to the DDC group. Baseline characteristics were well balanced between the two groups, with no statistically significant differences, both before and after IPTW (**Table 1**). Before IPTW, among the 12 prespecified baseline covariates, only 3 variables had standardized mean differences (SMDs) ranging from 0.10 to 0.15, while the SMDs of all remaining variables were below 0.10. After IPTW, covariate balance was further improved, with the SMDs of all variables reduced to below the conventional threshold of 0.10. This demonstrates that IPTW effectively eliminated baseline confounding between the two groups (**Supplementary Figure 1**).

**Table 1:** Comparison of baseline characteristics between the two patient groups.

Characteristics	Before IPTW				After IPTW			
	DDC group	DHDC group	P-value	SMD	DDC group	DHDC group	P-value	SMD
	(n = 64)	(n = 58)			(n = 122.1)	(n = 121.7)		
Gender			0.810	0.044			0.982	0.004
Male	54(84.4%)	48(82.8%)			101.0 (82.7%)	100.5 (82.5%)		
Female	10(15.6%)	10(17.2%)			21.1 (17.3%)	21.3 (17.5%)		
Age(y)			0.555	0.107			0.984	0.004
** ≤**60	33(51.6%)	33(56.9%)			65.5 (53.6%)	65.5 (53.8%)		
>60	31(48.4%)	25(43.1%)			56.6 (46.4%)	56.2 (46.2%)		
HBV infection			0.913	0.020			0.939	0.014
Yes	38(59.4%)	35(60.3%)			72.9 (59.7%)	71.8 (59.0%)		
No	26(40.6%)	23(39.7%)			49.2 (40.3%)	49.9 (41.0%)		
Cirrhosis			0.776	0.052			0.993	0.002
Yes	27(42.1%)	23(39.7%)			51.7 (42.3%)	51.4 (42.2%)		
No	37(57.8%)	35(60.3%)			70.4 (57.7%)	70.3 (57.8%)		
Child-Pugh Grade			0.717	0.066			0.979	0.005
A	31(48.4%)	30(51.7%)			61.8 (50.6%)	61.3 (50.4%)		
B	33(51.6%)	28(48.3%)			60.3 (49.4%)	60.4 (49.6%)		
ECOG PS			0.461	0.134			0.995	0.001
0	56(87.5%)	48(82.8%)			104.0 (85.1%)	103.6 (85.1%)		
1	8(12.5%)	10(17.2%)			18.2 (14.9%)	18.1 (14.9%)		
AFP (ng/mL)			0.961	0.009			0.996	0.001
**≤**400	24(37.5%)	22(37.9%)			45.7 (37.4%)	45.6 (37.4%)		
>400	40(62.5%)	36(62.1%)			76.4 (62.6%)	76.1 (62.6%)		
Tumor diameter(cm)			0.647	0.083			0.973	0.006
**≤**10	36(56.3%)	35(60.3%)			71.6 (58.6%)	71.7 (58.9%)		
>10	28(43.7%)	23(39.7%)			50.5 (41.4%)	50.0 (41.1%)		
Tumor number			0.485	0.127			0.983	0.004
**≤**3	38(59.4%)	38(65.5%)			76.4 (62.5%)	76.3 (62.7%)		
>3	26(40.6%)	20(34.5%)			45.8 (37.5%)	45.4 (37.3%)		
PVTT			0.882	0.027			0.970	0.007
Yes	24(37.5%)	21(36.2%)			44.5 (36.4%)	44.0 (36.1%)		
No	40(62.5%)	37(63.8%)			77.6 (63.6%)	77.8 (63.9%)		
BCLC stage			0.776	0.052			0.950	0.012
B	37(57.8%)	35(60.3%)			72.7 (59.6%)	73.2 (60.1%)		
C	27(42.2%)	23(39.7%)			49.4 (40.4%)	48.5 (39.9%)		
Extrahepatic metastasis			0.715	0.066			0.913	0.021
Yes	15(23.4%)	12(20.7%)			27.9 (22.8%)	26.8 (22.0%)		
No	49(76.6%)	46(79.3%)			94.2 (77.2%)	95.0 (78.0%)		

DHDC, DEB-TACE-HAIC combined with donafenib and camrelizumab; DDC, DEB-TACE combined with donafenib and camrelizumab; DEB-TACE, drug-eluting bead transarterial chemoembolization; HAIC, hepatic arterial infusion chemotherapy; HBV, hepatitis B virus; ECOG PS, Eastern Cooperative Oncology Group performance status;AFP, alpha-fetoprotein; PVTT, portal vein tumor thrombosis; BCLC, Barcelona Clinic Liver Cancer.

### Efficacy

#### Tumor responses

The median follow-up time was 18 months in the DHDC group versus 14 months in the DDC group. Tumor response outcomes of the two groups are summarized in **Table 2**. Before IPTW, according to the mRECIST, the DHDC group had significantly higher ORR (74.1% vs. 51.6%, *P* = 0.010) and DCR (91.4% vs. 75%, *P* = 0.017) compared with the DDC group. After IPTW, according to the mRECIST, the DHDC group had significantly higher ORR (72.5% vs. 53.4%, *P* = 0.036) and DCR (90.7% vs. 75.3%, *P* = 0.033) compared with the DDC group.

**Table 2:** Best tumor response based on mRECIST between the two groups.

Tumor responses	Before IPTW				After IPTW			
	DDC group	DHDC group	P-value	SMD	DDC group	DHDC group	P-value	SMD
	(n = 64)	(n = 58)			(n = 122.1)	(n = 121.7)		
CR	5(7.9%)	8(13.8%)			9.5 (7.8%)	15.6 (12.9%)		
PR	28(43.7%)	35(60.4%)			55.6 (45.6%)	72.6 (59.6%)		
SD	15(23.4%)	10(17.2%)			26.7 (21.9%)	22.2 (18.2%)		
PD	16(25%)	5(8.6%)			30.2 (24.7%)	11.3 (9.3%)		
ORR(CR+PR)	33(51.6%)	43(74.1%)	0.010	0.480	65.2 (53.4%)	88.2 (72.5%)	0.036	0.404
DCR(CR+PR+SD)	48(75%)	53(91.4%)	0.017	0.449	91.9 (75.3%)	110.4 (90.7%)	0.033	0.420

mRECIST, modified Response Evaluation Criteria in Solid Tumors, DHDC, DEB-TACE-HAIC combined with donafenib and camrelizumab; DDC, DEB-TACE combined with donafenib and camrelizumab; DEB-TACE, drug-eluting bead transarterial chemoembolization; HAIC, hepatic arterial infusion chemotherapy; CR, complete response; PR, partial response; SD, stable disease; PD, progressive disease; ORR, objective response rate; DCR, disease control rate.

#### Survival

**Figure 2:**
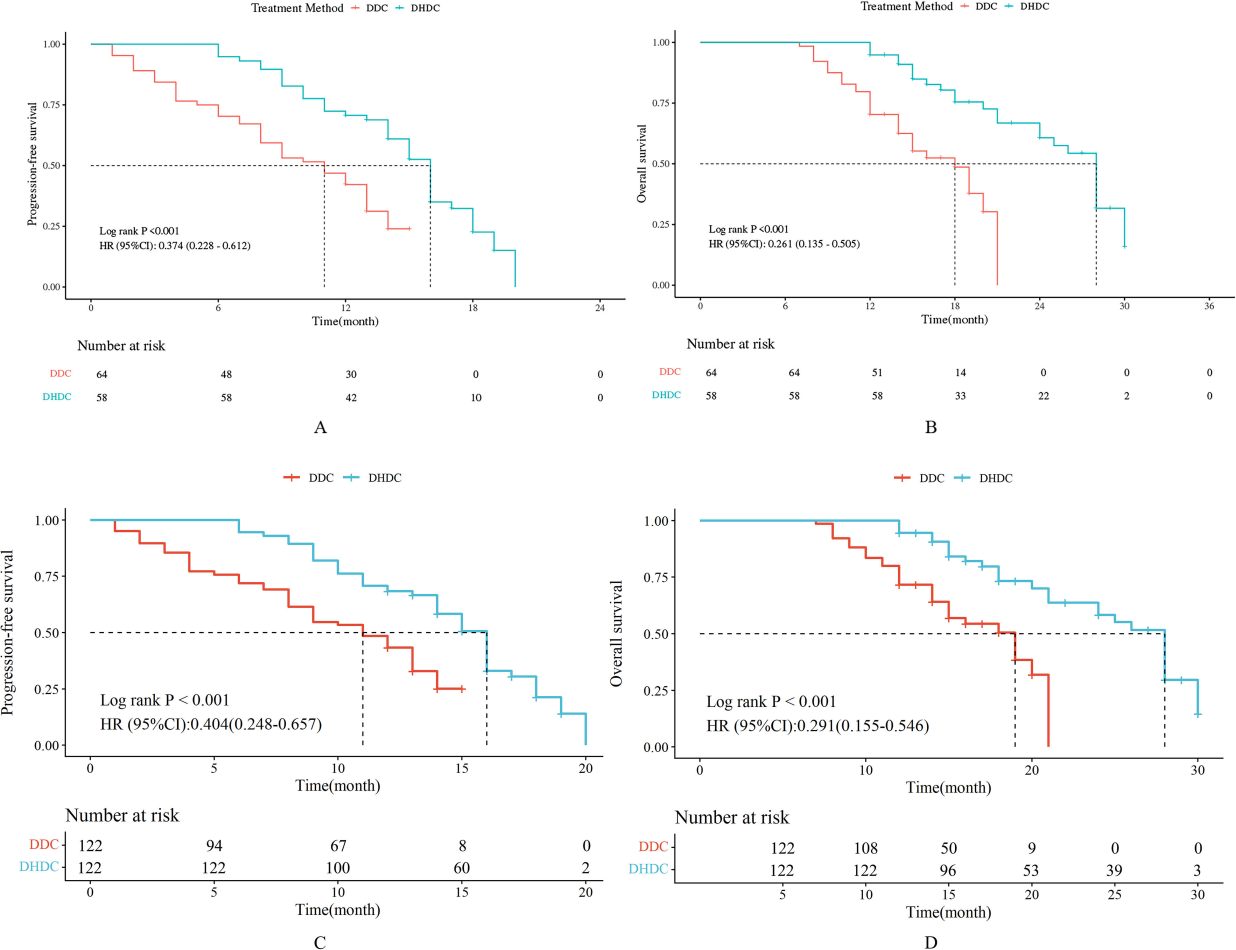
Kaplan-Meier survival curves of progression-free survival **(A)** and overall survival **(B)** in the two patient groups before IPTW. Kaplan-Meier curves of progression-free survival **(C)**, and overall survival **(D)** in the two patient groups after IPTW DHDC, DEB-TACE-HAIC Combined with Donafenib and Camrelizumab; DDC, DEB-TACE Combined with Donafenib and Camrelizumab; DEB-TACE, drug-eluting bead transarterial chemoembolization; HAIC, hepatic arterial infusion chemotherapy.

During follow-up, 25 of 58 patients (43.1%) in the DHDC group and 32 of 64 patients (50.0%) in the DDC group died. Before IPTW, the median PFS was significantly longer in the DHDC group than in the DDC group (16 months vs. 11 months; *P* < 0.001) (**Figure 2A**). Similarly, the median OS was significantly prolonged in the DHDC group compared with the DDC group (28 months vs. 18 months; *P* < 0.001) (**Figure 2B**). After IPTW, the median PFS was significantly longer in the DHDC group than in the DDC group (16 months vs. 11 months; *P* < 0.001) (**Figure 2C**). Similarly, the median OS was significantly prolonged in the DHDC group compared with the DDC group (28 months vs. 19 months; *P* < 0.001) (**Figure 2D**).

Multivariable Cox proportional hazards analysis demonstrated that PVTT (HR = 7.575; 95% CI, 1.721–33.333; *P* = 0.007) and extrahepatic metastasis (HR = 10.160; 95% CI, 4.737–21.791; *P* < 0.001) were independent risk factors for PFS, whereas treatment modality was identified as an independent protective factor for PFS (HR = 0.099; 95% CI, 0.052–0.189; *P* < 0.001). In addition, PVTT (HR = 8.319; 95% CI, 1.476–46.869; *P* = 0.016) and extrahepatic metastasis (HR = 21.465; 95% CI, 7.717–59.707; *P* < 0.001) were also independent risk factors for OS, while treatment modality remained an independent protective factor for OS (HR = 0.037; 95% CI, 0.013–0.105; *P* < 0.001) (**Tables 3**, **4**).

**Table 3:** Univariate and multivariate analysis of prognostic factors for PFS.

Variables	Univariate analysis		Multivariate analysis	
	HR (95%CI)	P-value	HR (95%CI)	P-value
Gender (Male vs Female)	0.860 (0.491 ~ 1.505)	0.597		
Age (>60 vs ≤60)	1.232 (0.798 ~ 1.901)	0.346		
HBV (Yes vs No)	1.156 (0.744 ~ 1.798)	0.518		
Cirrhosis (Yes vs No)	1.297 (0.841 ~ 2.000)	0.239		
Child-Pugh Grade (B vs A)	1.129 (0.735 ~ 1.736)	0.579		
ECOG PS (1 vs 0)	1.501 (0.855 ~ 2.634)	0.157		
AFP (>400 vs ≤400)	0.871 (0.560 ~ 1.355)	0.540		
Tumor diameter (>10 vs ≤10)	4.362 (2.770 ~ 6.870)	<0.001	1.300 (0.395 ~ 4.274)	0.666
Tumor number (>3 vs ≤3)	1.250 (0.806 ~ 1.937)	0.319		
PVTT (Yes vs No)	5.333 (3.375 ~ 8.427)	<0.001	7.575 (1.721 ~ 33.333)	0.007
BCLC stage (C vs B)	5.719 (3.597 ~ 9.095)	<0.001	0.926 (0.293 ~ 2.925)	0.896
Extrahepatic metastasis (Yes vs No)	7.178 (4.286 ~ 12.022)	<0.001	10.160 (4.737 ~ 21.791)	<0.001
Treatment modality (DHDC vs DDC)	0.374 (0.228 ~ 0.612)	<0.001	0.099 (0.052 ~ 0.189)	<0.001

HBV, hepatitis B virus; ECOG PS, Eastern Cooperative Oncology Group performance status;AFP, alpha-fetoprotein; PVTT, portal vein tumor thrombosis; BCLC, Barcelona Clinic Liver Cancer; DHDC, DEB-TACE-HAIC Combined with Donafenib and Camrelizumab; DDC, DEB-TACE Combined with Donafenib and Camrelizumab; DEB-TACE, drug-eluting bead transarterial chemoembolization; HAIC, hepatic arterial infusion chemotherapy; HR, hazard ratio; CI, confidence interval; PFS, progression-free survival.

**Table 4:** Univariate and multivariate analysis of prognostic factors for OS.

Variables	Univariate analysis		Multivariate analysis	
	HR (95%CI)	P-value	HR (95%CI)	P-value
Gender (Male vs Female)	0.959 (0.482 ~ 1.909)	0.906		
Age (>60 vs ≤60)	1.005 (0.595 ~ 1.697)	0.985		
HBV (Yes vs No)	1.466 (0.850 ~ 2.529)	0.169		
Cirrhosis (Yes vs No)	1.081 (0.638 ~ 1.833)	0.772		
Child-Pugh Grade (B vs A)	0.942 (0.559 ~ 1.589)	0.824		
ECOG PS (1 vs 0)	1.318 (0.692 ~ 2.509)	0.401		
AFP (>400 vs ≤400)	1.098 (0.640 ~ 1.881)	0.735		
Tumor diameter (>10 vs ≤10)	6.126 (3.434 ~ 10.925)	<0.001	2.003 (0.433 ~ 9.275)	0.374
Tumor number (>3 vs ≤3)	1.216 (0.712 ~ 2.078)	0.474		
PVTT (Yes vs No)	6.009 (3.439 ~ 10.498)	<0.001	8.319 (1.476 ~ 46.869)	0.016
BCLC stage (C vs B)	7.457 (4.050 ~ 13.730)	<0.001	0.419 (0.101 ~ 1.738)	0.231
Extrahepatic metastasis (Yes vs No)	7.964 (4.547 ~ 13.949)	<0.001	21.465 (7.717 ~ 59.707)	<0.001
Treatment modality (DHDC vs DDC)	0.261 (0.135 ~ 0.505)	<0.001	0.037 (0.013 ~ 0.105)	<0.001

HBV, hepatitis B virus; ECOG PS, Eastern Cooperative Oncology Group performance status;AFP, alpha-fetoprotein; PVTT, portal vein tumor thrombosis; BCLC, Barcelona Clinic Liver Cancer; DHDC, DEB-TACE-HAIC Combined with Donafenib and Camrelizumab; DDC, DEB-TACE Combined with Donafenib and Camrelizumab; DEB-TACE, drug-eluting bead transarterial chemoembolization; HAIC, hepatic arterial infusion chemotherapy; HR, hazard ratio; CI, confidence interval; OS, overall survival.

#### Subgroup analysis of PFS and OS

**Figure 3:**
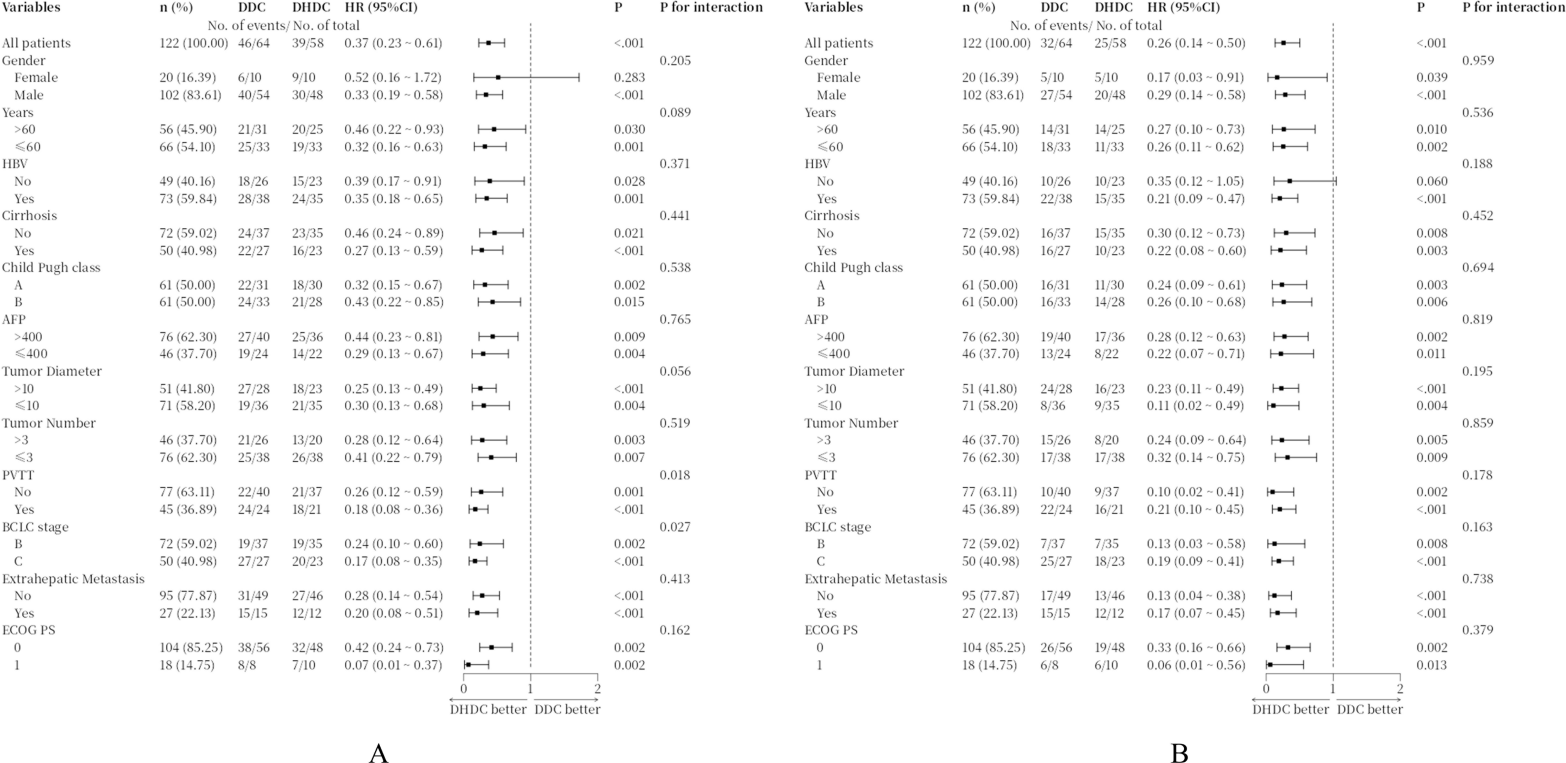
Forest plot of subgroup analysis for PFS **(A)** and OS **(B)** HBV, hepatitis B virus; ECOG PS, Eastern Cooperative Oncology Group performance status;AFP, alpha-fetoprotein; PVTT, portal vein tumor thrombosis; BCLC, Barcelona Clinic Liver Cancer; DHDC, DEB-TACE-HAIC Combined with Donafenib and Camrelizumab; DDC, DEB-TACE Combined with Donafenib and Camrelizumab; DEB-TACE, drug-eluting bead transarterial chemoembolization; HAIC, hepatic arterial infusion chemotherapy; HR, hazard ratio; CI, confidence interval; PFS, progression-free survival; OS, overall survival.

Prespecified subgroup analyses were performed for PFS (**Figure 3A**) and OS (**Figure 3B**) according to sex, age, ECOG PS, HBV infection status, Child–Pugh class, AFP level, presence of cirrhosis, BCLC stage, PVTT, extrahepatic metastasis, tumor size, and tumor number. The results demonstrated that the DHDC group consistently exhibited significant clinical benefits in both PFS and OS and showed superior outcomes compared with the DDC group across most clinical subgroups. However, these results are exploratory and should be interpreted with caution due to the small sample size within each subgroup. Further analyses suggested that the quadruple DHDC regimen was associated with particularly pronounced survival benefits in male patients, those with HBV infection, tumor number >3, cirrhosis, PVTT, BCLC stage C disease, extrahepatic metastasis, and tumor diameter >10 cm. These findings should be interpreted as preliminary, and larger, randomized controlled trials are needed to confirm the potential benefits of individualized treatment strategies for patients with distinct clinical characteristics.

#### Safety

Treatment-related AEs are detailed in **Table 5**. No treatment-related deaths were reported in this study. Among adverse events of all grades, the incidence of nausea and vomiting was significantly higher in the DHDC group (*P* < 0.05), while there was no statistically significant difference in the incidence of other AEs (*P* > 0.05). There was no significant difference in the incidence of grade 3 or higher AEs between the two groups (*P* > 0.05). Notably, no grade 4 or 5 AEs occurred in either group. The most common AEs included fever, abdominal pain, as well as elevated aspartate aminotransferase (AST) and alanine aminotransferase (ALT) levels.

**Table 5:** Comparison of treatment-related adverse events between the two patient groups.

Adverse events	Any grade			Grade≥3		
	DHDC group (n = 58)	DDC group (n = 64)	P	DHDC group (n = 58)	DDC group (n = 64)	P
Fever	46 (79.3%)	48 (75.0%)	0.572	0	0	-
Abdominal pain	41 (70.7%)	39 (60.9%)	0.258	0	0	-
Nausea	36 (62.1%)	26 (40.6%)	0.018	0	0	-
vomiting	31	19	0.008			
	(53.4%)	(29.7%)				
Hand-foot skin reaction	33 (56.9%)	38 (59.4%)	0.782	2 (3.4%)	2 (3.1%)	1.000
Alopecia	8 (14.8%)	6 (9.3%)	0.444	0	0	-
Rash	11 (20.4%)	15 (23.4%)	0.547	0	0	-
Diarrhea	20 (34.5%)	22 (34.4%)	0.990	0	0	-
Hyperbilirubinemia	31 (53.4%)	36 (56.3%)	0.756	4 (6.9%)	3 (4.7%)	0.707
Elevation of AST	38 (65.5%)	45 (70.3%)	0.571	5 (8.6%)	4 (6.3%)	0.878
Elevation of ALT	41 (70.7%)	44 (68.8%)	0.816	2 (3.4%)	3 (4.7%)	1.000
leukopenia	28 (48.3%)	30 (46.9%)	0.877	4 (6.9%)	7 (9.4%)	0.644
Thrombocytopenia	21 (36.2%)	24 (37.5%)	0.882	3 (5.2%)	4 (6.3%)	1.000
Proteinuria	22 (37.9%)	21 (32.8%)	0.555	1 (1.7%)	0	0.475
Hypertension	17 (29.3%)	22 (34.3%)	0.549	2 (3.4%)	3 (4.7%)	1.000
Weight Loss	19 (32.8%)	16 (25%)	0.344	0	0	-
Fatigue	21 (36.2%)	20 (31.3%)	0.759	0	0	-
Decreased appetite	41 (70.7%)	25 (39.1%)	0.563	0	0	-
Hypothyroidism	12 (20.7%)	17 (26.6%)	0.447	2 (3.4%)	2 (3.1%)	1.000
Gastrointestinal bleeding	2 (3.4%)	3 (4.7%)	1.000	0	0	-
RCCEP	17 (29.3%)	21 (32.8%)	0.677	1(1.7%)	0	0.475

DHDC, DEB-TACE-HAIC Combined with Donafenib and Camrelizumab; DDC, DEB-TACE Combined with Donafenib and Camrelizumab; DEB-TACE, drug-eluting bead transarterial chemoembolization; HAIC, hepatic arterial infusion chemotherapy; AST, aspartate aminotransferase; ALT, alanine aminotransferase; RCCEP, reactive cutaneous capillary endothelial proliferation.

Dose reduction of donafenib was required in 3 (5.2%) patients in the DHDC group and 2 (3.1%) in the DDC group. In addition, temporary interruption of camrelizumab due to adverse events was observed in 1 (1.7%) and 1 (1.6%) patient in the DHDC and DDC groups, respectively, and no patients required permanent discontinuation.

## Discussion

In this multicenter retrospective cohort study, we compared the efficacy and safety of the quadruple DHDC regimen with the triple DDC regimen in patients with uHCC. The DHDC regimen demonstrated significant advantages in tumor control and survival outcomes, with higher ORR and DCR and prolonged PFS and OS. Multivariable analysis identified PVTT and extrahepatic metastasis as independent adverse prognostic factors, while DHDC treatment was an independent protective factor. Subgroup analyses showed consistent benefits, particularly in patients with high tumor burden or aggressive disease. Although gastrointestinal adverse events were slightly more frequent in the DHDC group, they were mild and manageable. Overall, the DHDC strategy showed a favorable efficacy–safety profile. The survival benefit of the DHDC regimen is likely attributable to the synergistic effects of intensified locoregional and systemic therapies. While DEB-TACE provides sustained embolization and local tumor control, HAIC enables high local drug concentration and compensates for incomplete embolization, particularly in patients with high tumor burden (26).

DEB-TACE induces localized hypoxia within tumor tissue, which leads to upregulation of vascular endothelial growth factor (VEGF). This hypoxia-driven increase in VEGF not only promotes angiogenesis and contributes to local tumor recurrence (27), but also disrupts leukocyte–endothelial interactions, thereby impairing immune-cell infiltration into the tumor microenvironment (28). Accordingly, blockade of the VEGF pathway has been shown to increase effector T-cell infiltration and enhance the efficacy of immunotherapy. In addition, DEB-TACE can promote the release of tumor-associated antigens and proinflammatory cytokines and induce immunogenic cell death, thereby amplifying antitumor immune responses (29). Donafenib, through VEGF inhibition, attenuates hypoxia-induced angiogenesis following TACE and exerts both antiangiogenic and antiproliferative effects, while also contributing to immune modulation. Camrelizumab further restores antitumor immunity by reversing PD-1–mediated immune suppression. Notably, chemotherapeutic agents administered via HAIC have been shown to promote intratumoral infiltration of CD8^+^ T cells and favorably remodel the tumor immune microenvironment, thereby enhancing the antitumor activity of immune checkpoint blockade (30). Collectively, DEB-TACE and HAIC can reshape the tumor immune microenvironment and synergize with immunotherapy (31), forming a coordinated antitumor paradigm characterized by locoregional tumor debulking, targeted pathway inhibition, and immune activation.

For uHCC, treatment strategies have progressively shifted toward the integration of locoregional therapies (TACE or HAIC) with targeted therapy and immunotherapy. Several studies have shown that TACE–HAIC combined with targeted agents and immune checkpoint inhibitors can improve survival outcomes; however, the magnitude of benefit remains heterogeneous. Some studies have reported improved survival with quadruple therapy compared with triple regimens (32),whereas others have demonstrated favorable tumor responses with combination strategies (23) or comparable survival outcomes between quadruple and triple regimens, with some suggesting potential advantages of HAIC-based triple therapy (33–35) In addition, treatment sequencing may influence outcomes, with HAIC-first approaches showing more favorable tumor response and survival benefits in certain settings (36). Taken together, whether quadruple therapy confers a significant advantage over triple therapy in terms of tumor response and survival outcomes in patients with uHCC remains to be further validated. Notably, most existing studies are single-center and may be subject to selection bias. In contrast, our multicenter study further optimized locoregional therapy by incorporating DEB-TACE with HAIC and demonstrated favorable survival outcomes, supporting the potential value of intensified multimodal strategies.

Approximately 44%–62% of patients with HCC develop PVTT, which plays a critical role in prognostic stratification and clinical staging. Patients with HCC complicated by PVTT generally have a poor prognosis (37). Extrahepatic metastasis, most commonly involving the lungs in advanced HCC, is also associated with significantly shorter survival compared with disease confined to the liver (38). In the present study, multivariable Cox regression analysis identified both PVTT and extrahepatic metastasis as independent adverse prognostic factors, findings that are consistent with previous reports (39, 40).

Subgroup analyses demonstrated that the benefits of the DHDC regimen in terms of both PFS and OS were generally consistent across most clinical variables. Notably, more pronounced survival advantages were observed in male patients, those with HBV infection, a high tumor burden (defined as more than three tumors or a maximum tumor diameter exceeding 10 cm), coexisting cirrhosis, portal vein tumor thrombosis, extrahepatic metastasis, and BCLC stage C disease. These findings suggest that the DHDC regimen may be particularly suitable for patients with uHCC characterized by advanced stage and substantial tumor burden.

Although the DHDC quadruple regimen demonstrated substantial efficacy in the treatment of uHCC, its safety profile warrants careful consideration. In the present study, the incidence of nausea and vomiting was slightly higher in the DHDC group than in the DDC group; however, these events were predominantly low-grade, manageable with supportive care, and did not result in treatment discontinuation or severe toxicity. The increased frequency of gastrointestinal adverse events may be attributable to the incorporation of HAIC into the treatment regimen. Overall, the DHDC strategy achieved significant survival benefits while maintaining an acceptable safety profile.

Given the complexity of evaluating tumor response and the tumor microenvironment in the context of combination therapies, emerging imaging-based analytical approaches may provide additional value beyond conventional assessment criteria. For example, CT-based radiomics models can capture both global and intratumoral heterogeneity and have been used to predict treatment response and survival outcomes in patients with hepatocellular carcinoma receiving TACE combined with targeted therapy and immunotherapy. Notably, imaging-derived features have also been shown to correlate with the tumor immune microenvironment (41).

In addition, longitudinal imaging analyses have demonstrated that dynamic changes in body composition, including skeletal muscle and adipose tissue, are significantly associated with treatment response, survival outcomes, and treatment tolerability (42). These findings suggest that imaging can reflect not only tumor characteristics but also the overall host condition, which is increasingly recognized as an important determinant of therapeutic efficacy in intensified combination regimens.

In the context of multimodal treatment strategies such as DHDC, which integrate locoregional therapy with targeted therapy and immunotherapy, these advanced imaging approaches may provide a more comprehensive assessment of treatment-related heterogeneity, improve the accuracy of early efficacy evaluation, and facilitate more precise patient selection. The integration of imaging biomarkers into clinical decision-making may further promote the development of personalized treatment strategies.

Several limitations of this study should be acknowledged. First, the retrospective design is inherently subject to selection bias during patient enrollment. Second, the relatively limited sample size may have reduced the statistical power, and findings from certain subgroup analyses require validation in larger cohorts. Third, the follow-up duration was insufficient to fully assess long-term survival outcomes and cumulative treatment-related toxicities. Future prospective studies with larger sample sizes and longer follow-up are warranted to further clarify the role of this intensified multimodal strategy in uHCC.

## Conclusion

In conclusion, the DHDC quadruple regimen was associated with improved tumor response rates, significantly prolonged survival, and an acceptable safety profile in patients with uHCC. These findings suggest that DHDC may represent a promising therapeutic strategy for uHCC. However, given that the study was conducted in China with a predominantly HBV-related uHCC population, these results may be most applicable to Chinese. Further validation through large-scale, prospective randomized controlled trials is needed to confirm the efficacy and safety of this regimen.

## Data Availability

The raw data supporting the conclusions of this article will be made available by the authors, without undue reservation.
