# Supplementary figures and images for: DEB-TACE-HAIC combined with donafenib and camrelizumab in the treatment of unresectable hepatocellular carcinoma: a multicenter retrospective study
Source: Front Immunol. 2026 May 29;17:1779170. doi: 10.3389/fimmu.2026.1779170 (PMC13260537; doi:10.3389/fimmu.2026.1779170)

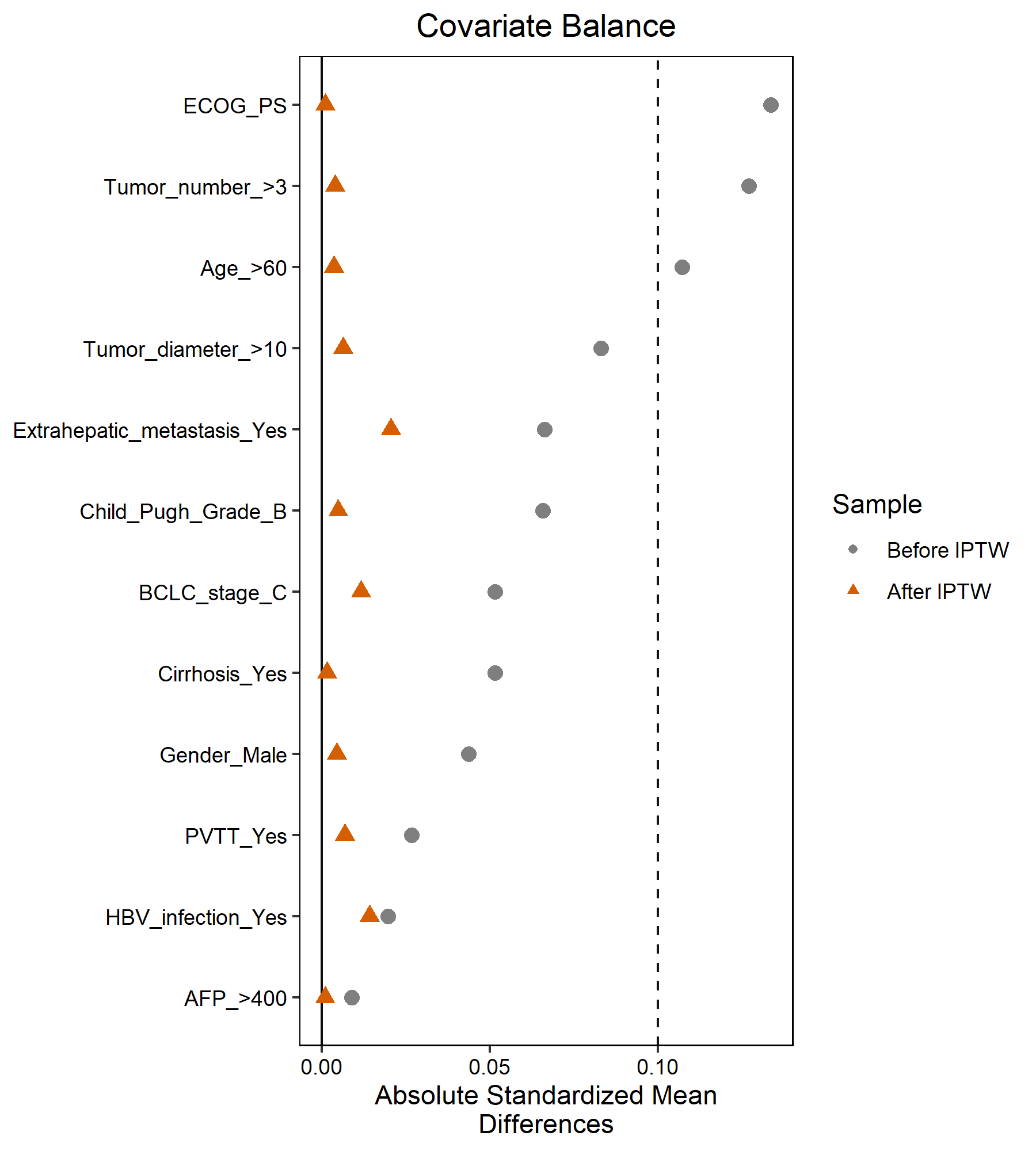

Supplement: Supplementary file 1 [file Image1.tiff]
